# Supplementary material for: Synthesis Antifreezing and Antidehydration Organohydrogels: One-Step In-Situ Gelling versus Two-Step Solvent Displacement
Source: Polymers (Basel). 2020 Nov 12;12(11):2670. doi: 10.3390/polym12112670 (PMC7696091; doi:10.3390/polym12112670)
Supplement: Supplementary file 1 [file polymers-12-02670-s001.pdf]

Supplementary Materials for

Synthesis Anti-freezing and Anti-dehydration Organohydrogels: One-Step In Situ Gelling *versus* Two-Step Solvent Displacement

Chun Li, Xiaobo Deng, Xiaohu Zhou*

College of Chemistry and Environmental Engineering, Shenzhen University, Shenzhen 518060, P. R. China

* Correspondence: xhzhou@szu.edu.cn (X.Z.)

Table S1. The density of different organic-water binary solvents.

Glycerol (v/v %)	ρ (g mL^{-1})	EG (v/v %)	ρ (g mL^{-1})
100	1.26	100	1.11
80	1.22	80	1.11
67	1.19	67	1.08
50	1.15	50	1.07
33	1.10	33	1.04
20	1.06	20	1.02
0	1.00	0	1.00

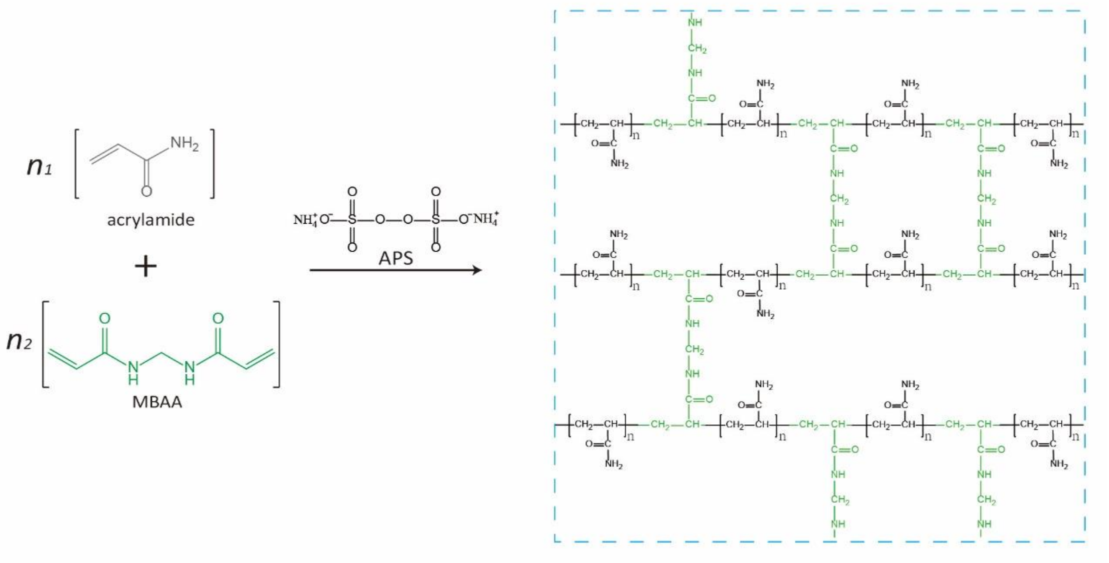

Figure S1. Schematic illustration of the polymerization of the PAAm matrix.

Table S2. Varied crosslinker (MBAA and PEGDA) and initiator (APS) contents in the PAAm gels with 5 wt% acrylamides (AAm).

No.	Glycerol (v/v %)	MBAA/ AAm (wt %)	PEGDA /AAm (wt %)	APS /AAm (wt %)	reaction condition	
1	80	0.5~5	-	10	UV	Too brittle.
2		0.5	-	5		Too brittle.
3		0.5	-	1		Unsmooth surface and brittle.
4		0.125	1.4	5		Compared to No. 2, the modulus increased while the tensile strain decreased.
5		0.3	-	5		Organohydrogel with moderate mechanical performance.
6		0.075	0.84	5		Compared to No. 5, the modulus increased while the tensile strain decreased.
7		0.3	-	2		Unsmooth surface.
8		0.2	-	5		Too sticky and soft to take out from the mould.
9		0.2	-	10		Too sticky and soft to take out from the mould.
10		0.1	-	5		Too sticky and soft to take out from the mould.
11	100	0.3	-	5	AAm and MBAA were first dissolved in glycerol, and then APS was added.	Organogel was formed before the APS completely dissolved in the precursor solution.
12					15 mL glycerol solution with AAm and MBAA mixed with 5 mL glycerol solution with APS (ice water bath).	Organogel was formed spontaneously without UV exposure. Too sticky and soft to take out from the mould.
13	0	0.3		5	UV	Hydrogel with moderate mechanical performance.
14	0	0.3		2		After reaction for one hour, there was still some unreacted precursor solution.

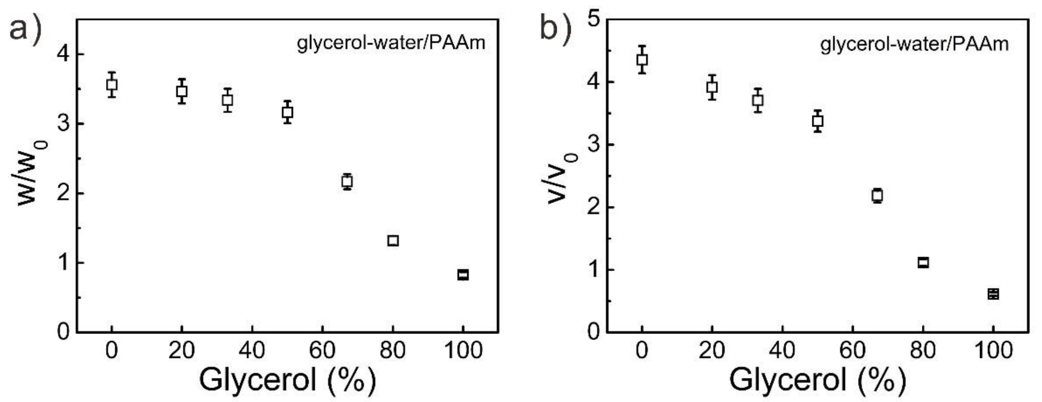

Figure S2. The swelling ratios of the PAAm hydrogel in the glycerol-water binary solvent with various glycerol fraction: a) swelling ratios by weight and b) swelling ratios by volume.

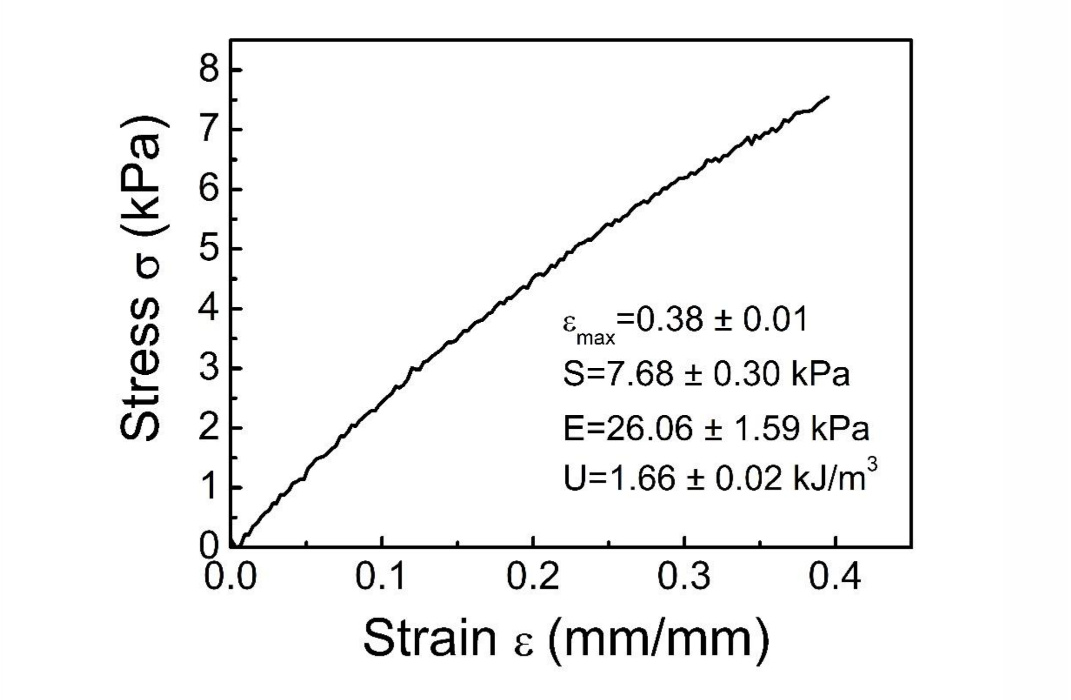

Figure S3. Mechanical properties of the PAAm hydrogels at equilibrium swollen state in water.

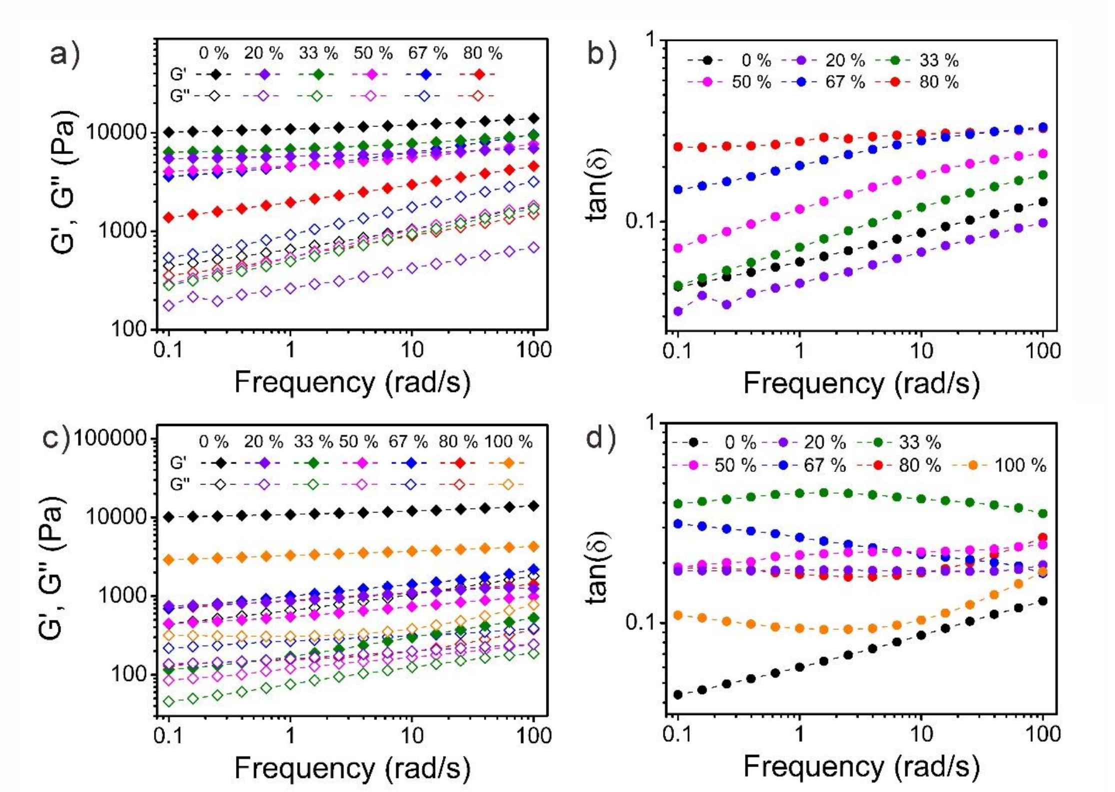

Figure S4. a, c) Modulus (*G'* and *G''*) and (b, d) loss factor (*tan*(δ)) as a function of frequency for the glycerol-based PAAm organohydrogels with different glycerol contents from (a, b) in situ gelling and (c, d) solvent displacement.

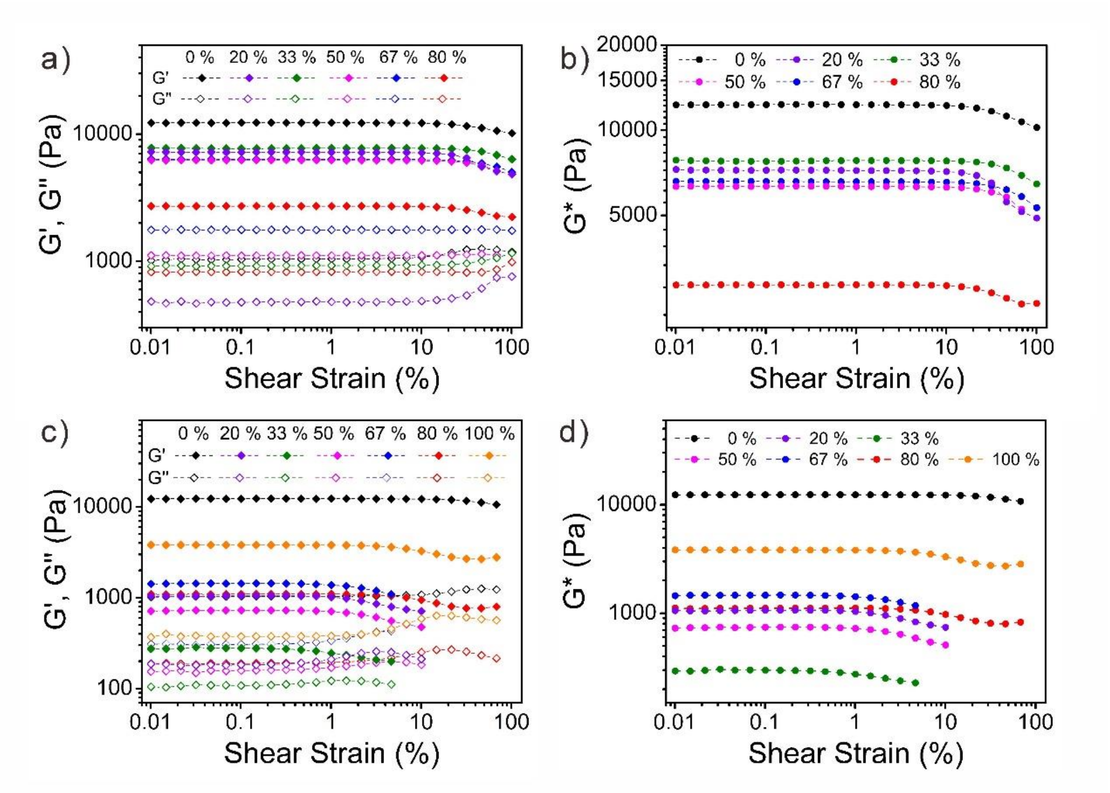

Figure S5. (a, c) Modulus (*G'* and *G''*) and (b, d) complex modulus (*G**) as a function of shear strain for the glycerol-based PAAm organohydrogels with different glycerol contents from (a, b) in situ gelling and (c, d) solvent displacement.

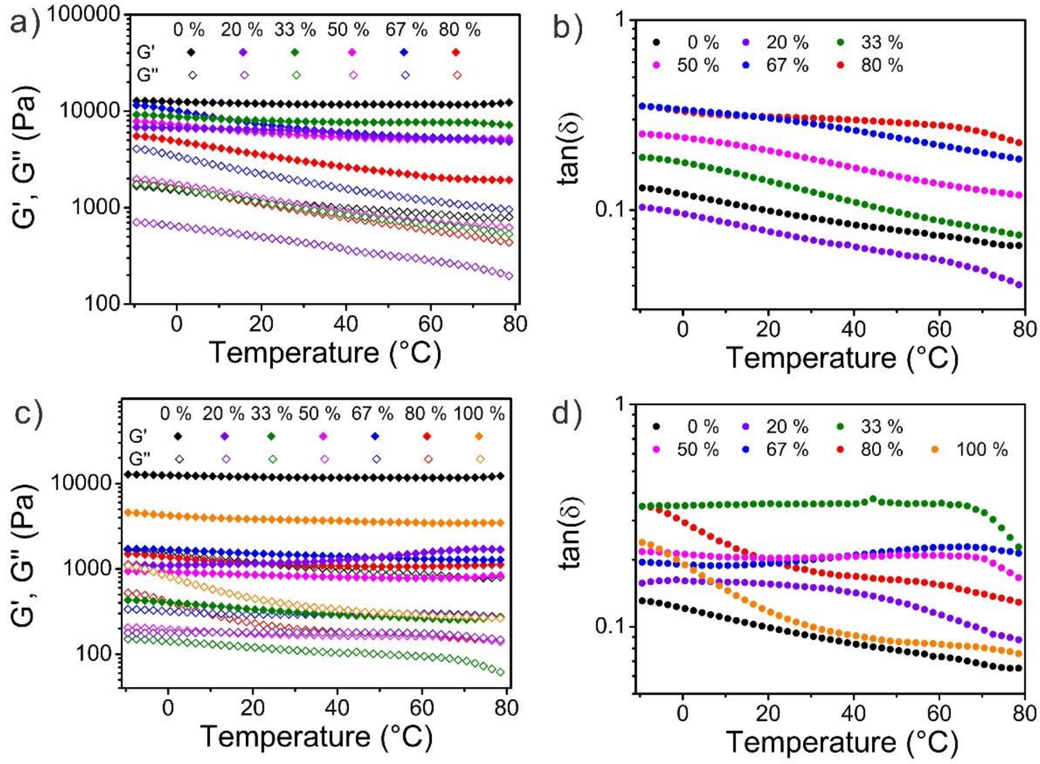

Figure S6. a, c) Modulus (*G'* and *G''*) and (b, d) loss factor (*tan(δ)*) as a function of temperature for the glycerol-based PAAm organohydrogels with different glycerol contents from (a, b) in situ gelling and (c, d) solvent displacement.

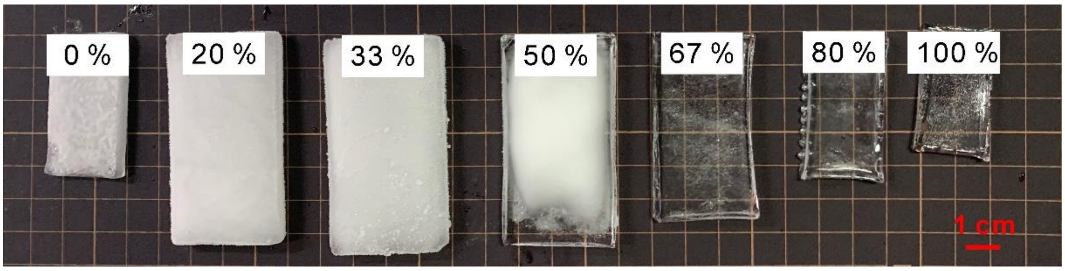

Figure S7. The digital photograph of the PAAm gels from the solvent displacement after stored at -40 ºC for 24 hours.

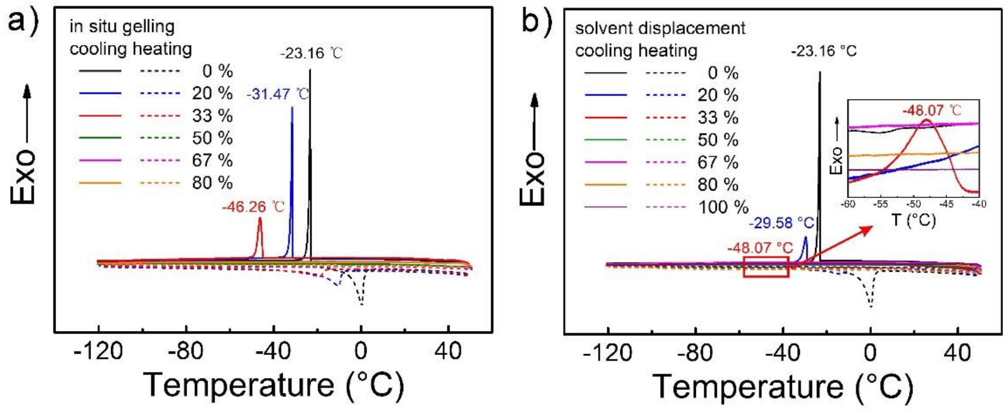

Figure S8. The experimental DSC results of the PAAm gels with various glycerol contents from a) in situ gelling and b) solvent displacement.

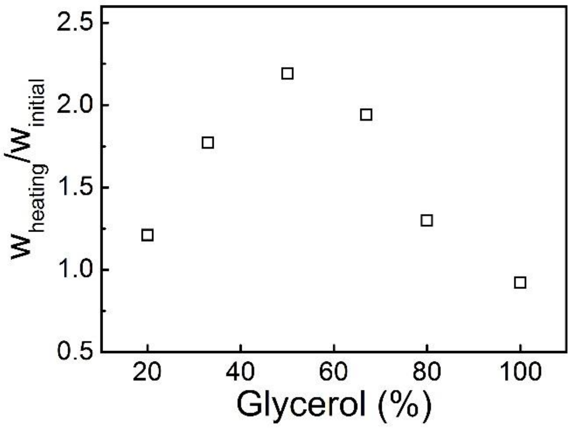

Figure S9. The remaining weight ratio of the PAAm gels after heating to the initial PAAm hydrogels.

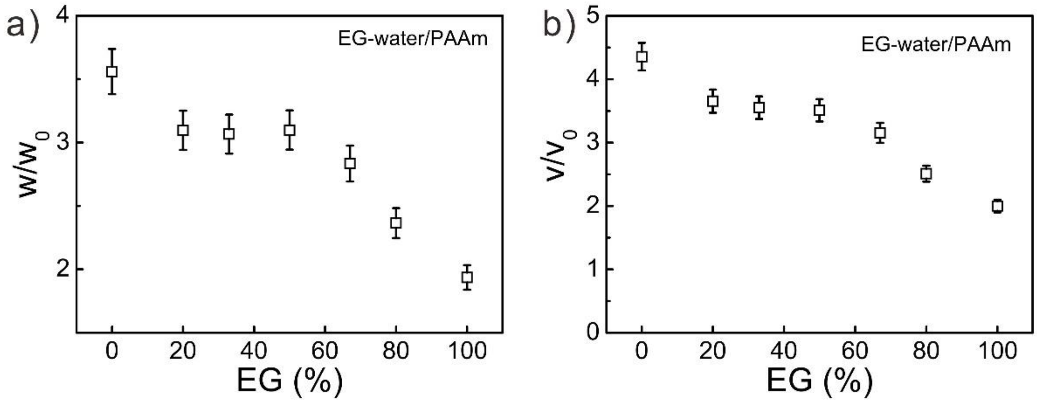

Figure S10. The swelling ratios of the PAAm hydrogel in the EG-water binary solvent with various EG fraction: a) swelling ratios by weight and b) swelling ratios by volume.

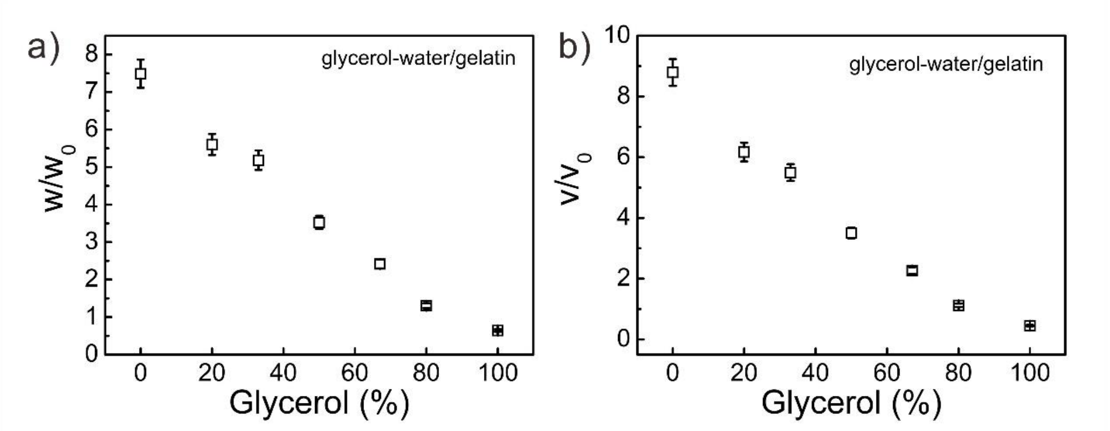

Figure S11. The swelling ratios of the gelatin hydrogel in the glycerol-water binary solvent with various glycerol fraction: a) swelling ratios by weight and b) swelling ratios by volume.
